# Fracture of the Acromion Process

**Published:** 1861-06

**Authors:** 


IV. C. C. O., aged 25; applied for relief May 8th. The evening previous, he received an injury from the fall of a tackle block, thirty feet, striking him upon the left shoulder. He suffered considerable pain, and the parts presented upon examination, a good deal of swelling and ecchymosis, and a noticeable flattening of the deltoid. After a complete examination, fracture of the acromion process was diagnosticated.

It often happens, said the Professor, that the surgeon may hesitate respecting the existence of fracture of this character, and a dislocation into the axilla—the physical signs apparent to the eye, in both cases are identical, so that when the shoulder is contused and swollen by the injury, as at present, the diagnosis is difficult and delicate. This may be simplified by the following points:

1st, In *dislocation* the deltoid can be crowded under the acromion.

2d, All normal conditions are impeded or abolished.

3d, The limb is longer than natural, and we feel for the head of the bone, in the axilla.

In *fracture* we may detect crepitation with care, and the movements of the arm are not restricted. A simple and easily discovered symptom, will always enable you to recognize this fracture with certainty, whether swelling exists or not:—*by raising the arm* with the forearm flexed, we *correct* the flattening and depressibility of the shoulder, restore its contour, determine crepitus, whilst the *deformity is re-produced* at once, when the limb is left unsupported.

